# Comparison of intensity modulated radiotherapy (IMRT) with intensity modulated particle therapy (IMPT) using fixed beams or an ion gantry for the treatment of patients with skull base meningiomas

**DOI:** 10.1186/1748-717X-7-44

**Published:** 2012-03-22

**Authors:** Katsura Kosaki, Swantje Ecker, Daniel Habermehl, Stefan Rieken, Oliver Jäkel, Klaus Herfarth, Jürgen Debus, Stephanie E Combs

**Affiliations:** 1 Department of Radiology, Nagoya City University Hospital, Nagoya, Japan; 2 Heidelberg Ion Therapy Center (HIT), Heidelberg, Germany; 3 Department of Radiation Oncology, University Hospital of Heidelberg, Im Neuenheimer Feld 400, 69120 Heidelberg, Germany; 4 Department of Medical Physics, German Cancer Research Center (dkfz), Heidelberg, Germany

**Keywords:** Plan comparison, Photon IMRT, Carbon ions, Protons, Gantry

## Abstract

**Background:**

To examine the potential improvement in treatment planning for patients with skull base meningioma using IMRT compared to carbon ion or proton beams with and without a gantry.

**Methods:**

Five patients originally treated with photon IMRT were selected for the study. Ion beams were chosen using a horizontal beam or an ion gantry. Intensity controlled raster scanning and the intensity modulated particle therapy mode were used for plan optimization. The evaluation included analysis of dose-volume histograms of the target volumes and organs at risk.

**Results:**

In comparison with carbon and proton beams only with horizontal beams, carbon ion treatment plans could spare the OARs more and concentrated on the target volumes more than proton and photon IMRT treatment plans. Using only a horizontal fixed beam, satisfactory plans could be achieved for skull base tumors.

**Conclusion:**

The results of the case studies showed that using IMPT has the potential to overcome the lack of a gantry for skull base tumors. Carbon ion plans offered slightly better dose distributions than proton plans, but the differences were not clinically significant with established dose prescription concepts.

## Introduction

Treatment of skull base tumors is a challenge for the radiation oncologist. Optimization of dose distributions to complex target volumes has been a main goal over the last decades, and modern photon techniques such as Intensity Modulated Radiotherapy (IMRT) have significantly improved treatment of base of skull tumors. Histological subtypes in the skull base region include highly malignant tumors such as high-grade hemangiopericytomas, squamous cell carcinomas or sarcomas, but also benign lesions, such as meningiomas. In these patients, high local doses for tumor control are also required, however, also sparing of normal tissue to prevent treatment-related side effects impairing quality of life is of high importance.

Several publications reported comparison studies in the head and neck region and the central nervous system using proton or carbon ion therapy as well as conventional and/or IMRT [1-8]. In photon treatments, a gantry is standard today and is necessary to obtain good dose distributions and conformity and to avoid high doses to organs at risk (OAR). IMRT can create distributions with higher concentration to be desired target volumes and to spare OARs. Several comparative studies using protons or ions showed a potential superiority over photons, especially for larger target volumes [6]. This is mostly due to the distinct characteristics of particle beams: Particle therapy has physical advantages with a sharp increase of dose in a well-defined depth (Bragg peak) and a rapid dose falloff beyond that maximum.

Protons have been used for treatment of meningiomas. Combined proton and photon radiotherapy was published from several institutions [9-12].

Experience with carbon ion radiotherapy has been acquired in the past mainly using horizontal beam lines. Excellent dose distributions can be achieved for numerous clinical cases using only horizontal beams. However the freedom to apply the beam on a gantry that rotates around the patient is expected to offer significant advantages, especially for certain anatomical regions, such as gastrointestinal tumors, paraspinal tumors, but also skull base lesions, as pointed out in Jäkel and Debus [13]. There is an extreme variation of density in these areas. These heterogeneities affect the energy distribution in the beam, e.g. a bone, decreases the particle's physical range as an air cavity extends the physical range as compared to water [14]. One of the advantages of a gantry is that it is possible to choose beam angles which pass through more homogeneous tissue, thus avoiding or reducing range uncertainties.

The Heidelberg Ion Therapy Center (HIT) started clinical operation in November 2009. At HIT, 3 treatment rooms, two with a horizontal fixed beam and one with a carbon ion and proton gantry, are available for treatment.

In the present study, we performed a comparative planning study for complex skull base tumors focusing on meningioma patients evaluating IMRT, protons and carbon ions with and without a gantry in an institutional *"real-time szenario"*. The IMPT mode is available at HIT and we adopted this mode for the calculation. The focus was put on evaluation of dose distributions, and DVH analysis with special respect to OAR.

## Materials and methods

Five patients originally treated with photon IMRT were selected for this comparative study. All patients were diagnosed with benign menigioma (Table 1). The mean age of the patients at the start of photon IMRT was 58.4 years (range 39-81 years), 3 males and 2 females. No patient had a history of former irradiation. IMRT was applied with a linear accelerator (Siemens, Erlangen, Germany) using the step-and-shoot technique in four patients and with helical Tomotherapy in one patient. Patients were immobilized in supine position with a Scotch Cast™ (3 M, St. Paul MN, USA) mask system as published previously [15,16]. CT scans were performed for treatment planning with a 3-mm slice thickness. For target volume definition, additional examinations such as contrast-enhanced MRI (magnetic resonance imaging) as well as 68 Ga-Dotatoc PET were used. For the treatment plan comparison, ion beams were chosen using a horizontal beam, or an ion gantry. Two different optimization modes are available at HIT within the treatment planning system. In the single beam optimization mode, the beams are optimized independently towards homogeneous dose distributions that, when summed up, result in the desired distribution. Meanwhile all beams are optimized simultaneously in IMPT mode. In this study, the IMPT mode was used for optimization.

**Table 1:** Clinical features of five meningioma patients

**Patient No**.	Sex	Age (years)	Tumor site	Histopathology	Target volume (cm^3^)
1	Male	61	ethmoid sinus	WHO I	76.62

2	Female	45	left skull base	WHO I	54.7

3	Male	39	right skull base	WHO I	77.4

4	Female	81	skull base - left temporal lobe	WHO I	170.3

5	Male	66	left skull base	WHO I	17.0

### Target volume definition for different techniques and dose concepts

For benign meningiomas the gross tumor volume (GTV) is defined as the macroscopically visible tumor on contrast-enhanced imaging; a clinical target volume (CTV) of about 1-2 mm is added to allow for potential microscopic spread. A planning target volume (PTV) is added depending on the technique used and the known setup inaccuracies, between 1-3 mm.

At HIT, we have established target volume and dosing concepts for different radiation techniques depending on the histology, the necessary clinical target volume, the required dose and the beam quality. With photons, skull base menigniomas are treated with total doses of 52.2-57.6 Gy depending on the size of the lesion and the vicinity to OAR, in single doses of 1.8 Gy. Since protons are associated with an overall comparable RBE, the same dose and fractionation schemes are applied with photons. For carbon ions, due to the reduced beam broadening as well as the known higher RBE, slightly hypofractionated regimens (with 3 Gy E single doses) have been established within our clinical routine at HIT, based on the favourable clinical data obtained at GSI. To compare plans in our *"real time scenario"*, these dose and fractionation schedules were used in the present analysis.

For target volume definition, PTV margins at HIT include not only setup inaccuracy as for photons, but also range uncertainties. For example, for skull base tumors, commonly, a median PTV margin of 3 mm is used in our institution for protons and carbon ions.

### Treatment plans and delivery

**Figure 1:**
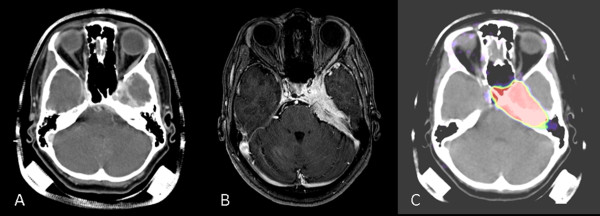
**An example of the pretreatment pictures**. (A) CT with contrast enhancement, (B) MRI with contrast enhancement and (C) 68 Ga-DOTATOC-PET for Case 2.

Figure 1 shows an example for the examinations used for treatment planning showing Case 2. Contouring of target volumes and OARs for particle therapy planning was performed with the Siemens Dosimetrist and Oncologist software (Siemens, Erlangen, Germany). On the other hand, photon IMRT treatments were carried out with two different treatment machines. Calculations for photon IMRT were based on inverse treatment planning with the Hi-ART Tomotherapy planning station version 2.2.1.55 (Tomotherapy Incorporated, Madison, WI, USA) and the Konrad System version 2.2.23 (Siemens, Erlangen, Germany). 6-MV photon beams were used for both plans. IMRT treatment planning was performed on the basis of an isocentric eight to nine-beam arrangement, with Konrad in the step-and-shoot technique.

The Treatment Planning System for particle therapy was performed by a commercial treatment planning system Syngo PT Planning (Siemens, Erlangen, Germany). Plan calculation was based on the treatment planning CT without contrast enhancement. For the OARs considered in this comparison study, the tolerance levels were based on the work of Emami et al [17]. The doses to OARs were set below maximum 60 Gy for the brainstem; 50 Gy for the optic chiasm; 50 Gy for the optic nerves; 45 Gy for the optic globe; 10 Gy for the lens; 32 Gy for the parotid gland. To compare these results directly, target structures and OARs contoured for the original photon IMRT plans were used also for the particle therapy plans. Two to three coplanar or noncoplanar beams were used for particle therapy treatment. The gantry rotation was not restricted in both coplanar and noncoplanar setup, but the treatment table was restricted to a range between 10 and 170 degrees within the provided software due to collision of the horizontal beam nozzle with the table. The couch and gantry angles of particle therapy plans were designed according to the restriction (Table 2). The maximum field size for both proton and carbon ion therapy at HIT is 20 by 20 cm. The pencil beams chosen for this study typically have a lateral full-width-at-half-maximum (FWHM) of 6 mm for carbon ion and 10 mm for proton treatment plans.

**Table 2:** Couch and gantry angles for patients

Patient **No**.	original photon IMRT	carbon/proton ion horizontal plan	proton gantry plan
1	helical Tomotherapy	(8, 90) (172, 90) (272, 90)	none

2	8 beam IMRT	(8, 90) (330, 90)	(8, 70) (8, 120)

3	8 beam IMRT	(172, 90) (210, 90)	(355, 240) (355, 270)

4	8 beam IMRT	(8,90) (335, 90)	none

5	8 beam IMRT	(8,90) (172, 90) (320, 90)	(0,150) (0, 305) (270, 60)

Case 2-5 were treated with a linear accelerator using step and shoot technique. (x, y) shows (couch angle, gantry angle)

Prescribed dose for original treatment planning with photon IMRT was tailored according to age, tumor volume and tumor location. Prescription dose for protons varied from 53.65 to 57.66 GyE (1.8 GyE to 1.86 GyE/Fr) since all patients were diagnosed benign meningiomas and there have been reports of good control of benign meningiomas with proton ion therapy [10,18,19]. However carbon ion therapy may play a great role in treating high risk meningiomas [20]. Based on our clinical concept for carbon ion treatments with the aim to perform a comparison in a "*real time szenario*", all carbon plans were prescribed at 60 GyE/20Fr (3 GyE/Fr; Table 3). The target volume was encompassed by the 95% isodose line in principle, but some areas where were adjacent to OARs were covered with 90% isodose line. However 90% and 95% isodose lines are very close in particle therapy plans.

**Table 3:** Dose prescripition for patients (original dose, proton dose, carbon ion dose)

**Patient No**.	original photon IMRT		carbon ion plan		proton plan	
	
	total dose	fraction	total dose	fraction	total dose	fraction
1	57.6 GyE	32 Fr	60 GyE	20 Fr	57.6 GyE	32 Fr

2	57.66 GyE	31 Fr	60 GyE	20 Fr	57.66 GyE	31 Fr

3	57.66 GyE	31 Fr	60 GyE	20 Fr	57.66 GyE	31 Fr

4	53.94 GyE	29 Fr	60 GyE	20 Fr	53.94 GyE	29 Fr

5	53.65 GyE	29 Fr	60 GyE	20 Fr	53.65 GyE	29 Fr

### Evaluation tools

The evaluation included analysis of dose-volume histograms of the target volumes and organs at risk. For each patient and each organ, a set of physical parameters was computed from the DVHs to assess the general characteristics of different techniques. The same dose constraints were adopted for optimization of the same patient's plan. Plans were also assessed by visual inspection of dose distributions.

## Results

Each patient was analyzed individually. PTV size data for each patient are reported in Table 1. The mean volume was 79.2 cm^3^, median 76.6 cm^3^, minimum 17.0 cm^3^, and maximum 170.3 cm^3^.

### Dose distributions and dose volume histograms for each case

**Figure 2:**
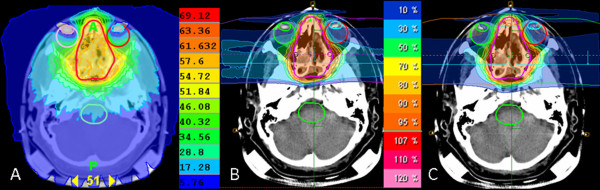
**Dose distribution in transverse plane for (A) photon IMRT, (B) carbon ion and (C) proton treatment planning techniques**. The same beam arrangements were used for carbon ion and proton plans. These plans consisted of two lateral beams and one cranial beam.

**Figure 3:**
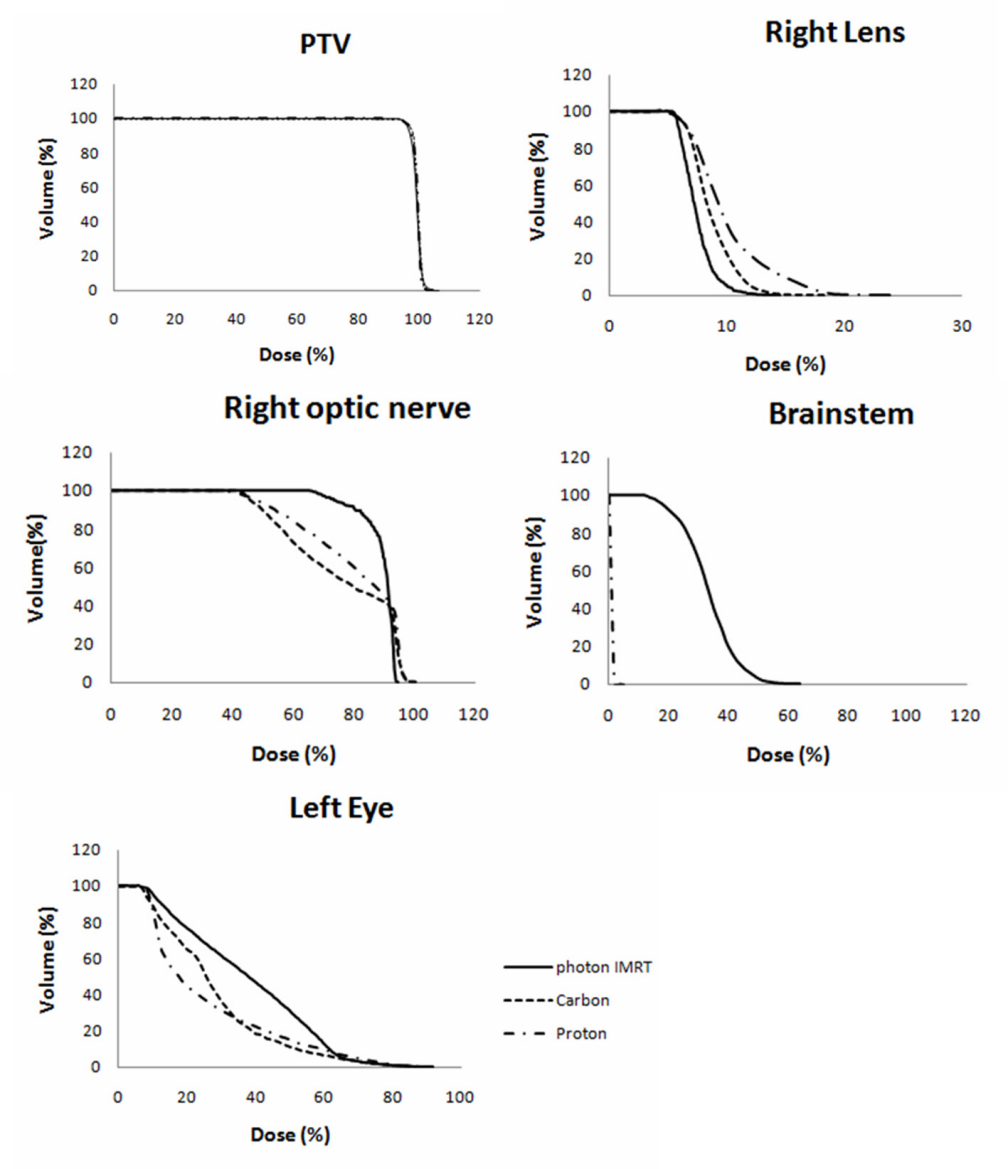
**Cumulative dose volume histograms for PTV, right lens, right optic nerve, brainstem and left eye for one Case 1 (given as a percentage of the prescribed target dose)**.

Figure 2 shows axial slices for photon IMRT, carbon ion and proton plans for Case 1. Figure 3 shows cumulative dose volume histograms for the PTV, brain stem, right lens, right optic nerve and left eye (given as a percentage of the prescribed target dose). The tumor was located at the center of the sphenoid wing and ethmoid sinus area. The IMRT plan was made with Tomotherapy and three beams were used for carbon ion and proton plan only with horizontal beams. Table 4 demonstrates the results of V_x _which were computed from the DVH curves to compare the characteristics of photon IMRT, carbon ion and proton. V_x _corresponds to the fractional volume irradiated to a percentage dose higher than x% [8]. Tomotherapy and carbon ion radiotherapy were superior to proton therapy regarding circumvention of both eye lenses. This is solely due to the horizontal beam direction chosen for proton and ion beams. Both particle therapies allowed a nearly complete avoidance of the brain stem as opposed to Tomotherapy which irradiated the brain stem with about 10-30% of the dose. The results of the DVH curves also demonstrated the results of the visual inspections. The curves of the PTV were nearly the same in all three radiotherapies. The right lens was well spared using Tomotherapy and carbon ion radiotherapy. The sparing of the brain stem was achieved only at high doses in the case of Tomotherapy, but also for low doses with proton therapy and complete sparing was achieved with carbon therapy.

**Table 4:** Results of the dose distribution for the PTV and OARs of case 1

Organ	Parameter	photon IMRT (%)	carbon ion (%)	proton (%)
PTV	V_90_	99.9	100.0	99.6

Right lens	V_10_	4.8	22.0	32.8

Right optic nerve	V_60_	100.0	73.2	83.1

Brainstem	V_40_	19.9	0	0

Left eye	V_40_	46.6	18.4	22.3

V_x _(corresponding to the fractional volume irradiated to a percentage dose higher than x%)

**Figure 4:**
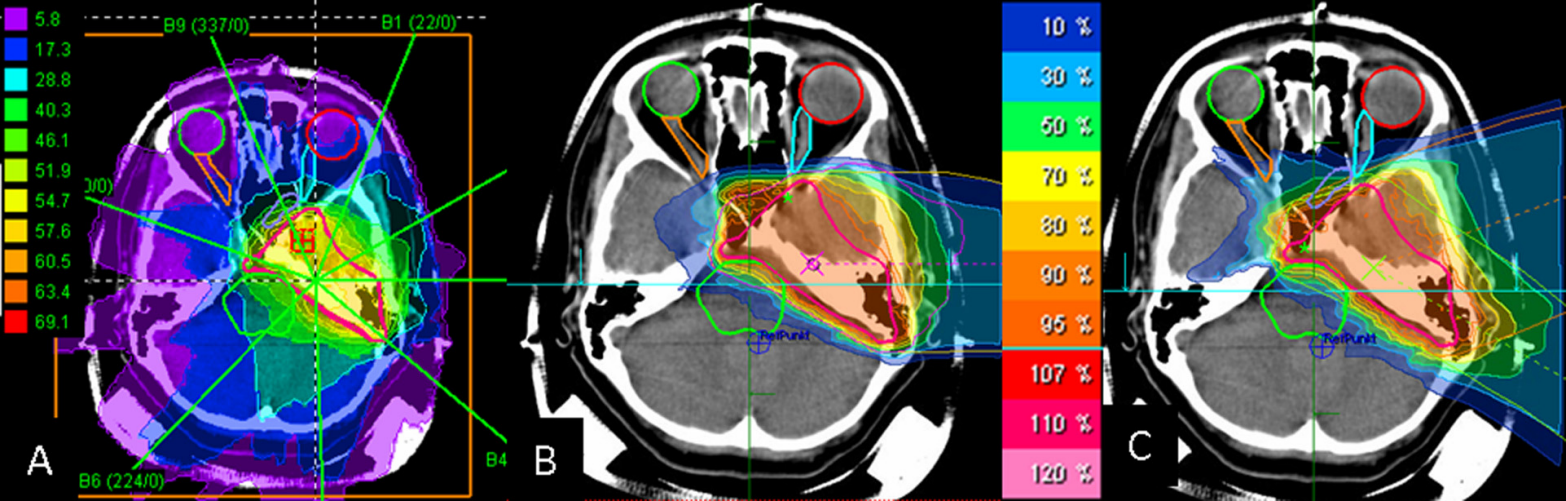
**Dose distributions for (A) photon IMRT plan, (B) proton plan with horizontal beams, (C) proton plan with gantry beams**.

The tumor locations of Case 2 and Case 3 were similar. Both meningiomas were attached to brain stem, one located on the left side of the brain stem and the other located on the right side of the brain stem. The resulting dose distributions of proton plans and photon IMRT plans for Case 2 are shown in Figure 4. They included at least one of the optic nerves. As the is tumor located one-sided, two beams were chosen from the same side of the tumor using protons. At the comparison of the dose-volume histograms between a gantry plan and a plan with horizontal beams, a gantry plan showed better dose sparing for some organs at risk in Case 2, but the differences were small. The dose-volume histograms for the OARs for Case 3 were almost comparable. The merit of a gantry for these cases was that we could choose beam angles avoiding air-filled cavities. These areas usually lead to substantial range uncertainties. For example, in the plan of Figure 4(B), both beams came from the left side and they were delivered with horizontal fixed beams. The effect of the air filled cavity leading to possible over- or under-shoot would appear around the brain stem. On the other hand, two beams came from left side but they were a little tilted to the anterior and posterior side using a gantry in Figure 4(C). One beam from the posterior side passed through the air filled cavity but possible overshoot would appear around the sphenoidal sinus area and not within the brain stem region. The PTV of Case 4 was 170.3 cm^3 ^and tumor was extended widely into the left eye area. It also included both optic nerves and a part of the left retina. Because the target was relatively large, it was difficult to place beams three dimensionally to cover this PTV while avoiding OARs at the same time. In this case, plans only with horizontal beams also could achieve satisfactory dose distributions particle therapy treatment. The dose-volume histogram curves of the PTV were nearly the same in all three radiotherapy szenarios in this case. In comparison with carbon and proton beams only with horizontal beams, the carbon ion treatment plan could spare the OAR much more compared to proton and photon IMRT treatment plans.

**Figure 5:**
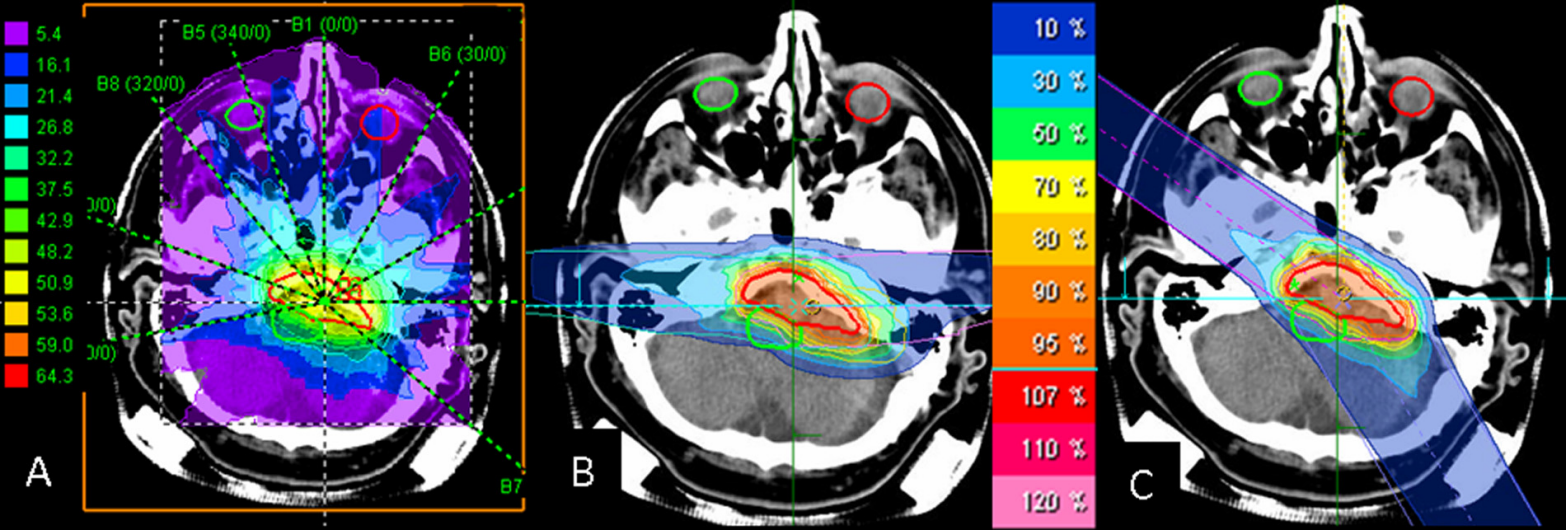
**Dose distributions for (A) photon IMRT plan, (B) proton plan with horizontal beams, (C) proton plan with gantry beams**.

In Case 5, the PTV for this patient was relatively small (17 cm^3^), it was adjacent to the brain stem but separated from other organs at risk. Therefore the brain stem was paid the most attention in this case. Demonstration plans were made to examine whether using a gantry had a benefit for proton planning. Figure 5 showed the dose distributions of three different plans for Case 5. We employed two beams for particle radiotherapy plans. One beam was placed in order to avoid the mastoid antrum which contains air. The other beam was placed on the cranial side. The photon IMRT plan was designed with nine beams. The PTV curves of three plans were almost the same. It was difficult to observe advantages of the gantry from the result of the dose-volume histograms in this case, as shown in Table 5. One advantage with a gantry was that it was easier to place beams to spare the mastoid antrum. IMRT plans were optimal, and were hardly improved by ion beams in this case.

**Table 5:** Results of the dose distribution for the PTV and OAR of case 5

Organ	Parameter	photon IMRT (%)	gantry plan(%)	horizontal plan (%)
PTV	V_90_	97.2	98.5	98.8

Brainstem	V_40_	25.6	20.5	21.4

## Discussion

In the present work we compared treatment planning using IMRT, protons and carbon ions for complex-shaped menigiomas in the skull base region. In most cases, IMRT provided excellent dose distributions, and only for certain OAR particle therapy showed a substantial benefit. Even for complex shapes a gantry was not essential in most cases. However, to avoid air-filled cavities in some clinical situations to reduce uncertainties, beam angles provided by a gantry are most likely to be highly superior. A quantitative analysis of the robustness of the treatment plans against these effects was beyond the scope of this work.

Compared to other intracranial tumors, skull base meningiomas often have complex shapes [21-27]. Particle therapy may be offered to patients with meniningiomas, especially as they are characterized by long-term survival, and minimization of treatment related side effects is of high importance, including neurocognitive sequelae [18-20,28]. Therefore, increasing dose conformality and reducing dose to normal tissue is of high importance. Particle therapy could offer highly conformal irradiation for complex-shaped intracranial meningiomas, since it is characterized by excellent dose conformity. In this study, two comparisons were examined. First, original photon plans and demonstration plans using proton and carbon ion were compared. Second, these comparison plans were made as well to examine the benefits of a gantry when skull base tumors were treated with particle therapy. Comparisons were carried out in terms of physical parameters derived from 3D dose distributions and DVH calculations. Since the aim of the study was to provide some rationaly as to which patients clearly benefit from a gantry as opposed to a horizontal beam (since at HIT, both possibilities are available) a *"real-time-szenario" *was chosen respecting the institutional dose and fractionation schemes for photons, protons and carbon ions. For example, due to the biological and physical characteristics of the carbon beam, carbon ions are applied with 3 Gy E single doses in our institution [20]. This includes not only dose and fractionation schemes, but also dose constraints and dose-volume-parameters used for treatment plan optimization.

In particle therapy planning, spot-scanning delivery methods were used and the IMPT mode was used for optimization. From a physical point of view, carbon ions show a sharper Bragg peak and less lateral scattering than protons, but also a significant dose in the fragmentation tail behind the peak [29]. In our comparison study, carbon ion plans could achieve better conformation of the dose to the target volumes than proton plans especially in the high dose areas and better sparing of dose to most of the organs at risk. But the differences were small and proton plans resulted in better sparing of some of the organs at risk as compared to photon IMRT. Concerning the additional improvement of dose concentration using carbon ions, however, there is no clear picture. Carbon beams show a higher dose behind the target because of the fragmentation tail. Therefore the low dose area around 30% isodose for carbon ions plans is larger in the distal region than for proton plans. Photon IMRT could also spare some organs at risk well in some cases, but the low-dose area in the photon IMRT plans were obviously much larger than in particle therapy plans. It should be kept in mind, however, that an additional benefit from carbon ions which is expected due to their radiobiological properties is not displayed in the treatment plan. E.g. a higher RBE is expected in the tumor as compared to the normal tissue, but a fixed RBE table is currently used for all tissues in the TPS. Additionally, is should be kept in mind that differences in pencil beam size can potentially contribute to differences in the treatment plan differences between ions themselves, but also compared to photons.

Baumert et al. reported that protons were generally better than photons at sparing dose to the OAR which was close to the target. But when they used only two fields to one patient, protons showed higher doses to surrounding OARs. A higher number of fields may result in a better conformity [5]. It was also experienced in our study. Compared to photon IMRT, small numbers of beams are employed for particle therapy. That means one beam has a big influence on the dose distribution and beam arrangements are very important. In making demonstration plans, we avoided to use an anterior beam to spare dose from sensitive OARs such as the eyes. Additionally an anterior field would have to pass through the oral cavity which poses large problems for proton and carbon ion calculations due to the large variability of position and interfaces between materials with large density differences.

The calculation results showed that the OARs which were far from targets were irradiated with lower mean doses in particle therapy than in photon IMRT. For the maximum dose, some cases showed higher doses in particle plans than in photon plans, possibly due to the use of only two or three radiation fields. Depending on the tumor location, even a third beam was not necessary. Lomax et al. performed a comparison study between IMRT and proton radiotherapy and nine beams were employed to compare the plans [2]. They used the identical beam geometry, dose constraints, importance factor, but changed dose-volume constraints depending on the plans. In this study, they reported the use of nine proton fields could well be sub-optimal in paranasal sinus area tumor, and the use of a smaller number of well chosen fields could achieve a similar level of target coverage and sparing of critical structures.

For facilities equipped with gantries, planning for dose delivery of protons and for carbon ions therapy has the same degrees of freedom as for the photon therapy. The aim of our study was to evaluate which cases are relatively easily accessible with horizontal fixed beam, and in which cases a gantry is really beneficial. In this study, the advantage of a gantry is not obvious. The first reason may be that most of the tumor shapes were irregular and complex and even if the gantry was used, it was difficult to find beam arrangements avoiding organs at risk while fully irradiating the target volumes at the same time. The second reason is probably that the IMPT optimization mode was adopted. IMPT plans can be delivered using the spot scanning system at HIT. Before making IMPT plans, we made demonstration plans with the single beam optimization (SBO) mode. Compared with these two different modes, the results were apparently better using IMPT mode than SBO mode. IMPT mode could improve the conformity inside the target volumes and spare the organs at risk which were in the vicinity of the tumors. Mizumoto et al. have reported the results of the three patients' treatment plans using non-coplanar beams in Tsukuba, Japan [30]. They concluded that non-coplanar proton beam therapy has advantages in selected patients. If the tumor extends mainly along the beam direction, the advantage of the gantry beams would be significant. The major difference of our study as compared to [30] is the treatment delivery system. The proton facility in Tsukuba used passive beam delivery, while HIT adopts active spot scanning beam delivery. In active beam delivery, the energy of the incoming beam is varied during the treatment [31]. Consequently IMPT can be accomplished. Because of the IMPT mode, it was considered that the advantage of the gantry with active methods seemed to be less than with passive methods. The results in this study showed that clinically satisfactory plans could be achieved by means of a horizontal beam line for skull base tumors with IMPT optimization [32] and a gantry does not lead to a clinically significant improvement in most cases. The role of uncertainties for both options is, however, not clear yet. That is to say, the IMPT mode has the potential to overcome the lack of the gantry for patients with skull base tumors.

## Conclusion

This study attempted to explore the differences of dose distribution between carbon ions and protons compared to photons in an institutional *"real-time szenario"*. Compared to photon IMRT, only marginal improvements may be seen at the required doses and dose distributions. For tumors requiring even higher doses, the additional benefit of a particle beam may be much greater, due to the reduction of integral dose. We also attempted to analyse the potential benefit of the gantry for skull base meningiomas. In the analysis of these five cases, carbon ion plans showed better dose conformation to the target volumes and sparing of OAR than proton plans in most of the cases. These differences were small and the advantage of carbon ions compared with protons is not always obvious. However, this work implied that the IMPT mode may offer treatment plans with improved quality for skull base tumors even if the facility doesn't have a gantry. This finding is likely to change for tumors located in the trunk of the body, e.g. gastrointestinal tumors or paraspinal tumors, where the access through highly sensitive OAR in combination with deeply located target volumes with horizontal beams may be much more difficult.

## Abbreviations

DVH: Dose Volume Histogram; Fr: Fractions; GyE: Gray Equivalent; HIT: Heidelberg Ion Therapy Center; IMPT: Intensity Modulated Particle Therapy; IMRT: Intensity Modulated Radiation Therapy; OAR: Organ at risk; PTV: Planning Target Volume; RBE: Relative Biological Effectiveness; SBO: Single Beam Optimization; TPS: Treatment Planning System; WHO: World Health Organization

## Competing interests

The authors declare that they have no competing interests.

## Authors' contributions

KK, SEC and JD have developed the study design. KK and SEC performed the patient selection, interpreted the data and drafted the manuscript. KK, SE, DH and SR conducted treatment planning. KK, SE and OJ decided beam arrangements for particle plans and analysis. SEC, KH made original photon IMRT plans. All authors read and approved the final manuscript.
